# Successful treatment of biofilm infections using shock waves combined with antibiotic therapy

**DOI:** 10.1038/srep17440

**Published:** 2015-12-10

**Authors:** Divya Prakash Gnanadhas, Monalisha Elango, S. Janardhanraj, C. S. Srinandan, Akshay Datey, Richard A. Strugnell, Jagadeesh Gopalan, Dipshikha Chakravortty

**Affiliations:** 1 Department of Microbiology and Cell Biology, Indian Institute of Science, Bangalore, India; 2 Centre for Biosystems Science and Engineering, Indian Institute of Science, Bangalore, India; 3 Department of Aerospace Engineering, Indian Institute of Science, Bangalore, India; 4 Department of Microbiology and Immunology, The Peter Doherty Centre for Infection and Immunity at The University of Melbourne, Australia

## Abstract

Many bacteria secrete a highly hydrated framework of extracellular polymer matrix on suitable substrates and embed within the matrix to form a biofilm. Bacterial biofilms are observed on many medical devices, endocarditis, periodontitis and lung infections in cystic fibrosis patients. Bacteria in biofilm are protected from antibiotics and >1,000 times of the minimum inhibitory concentration may be required to treat biofilm infections. Here, we demonstrated that shock waves could be used to remove *Salmonella*, *Pseudomonas* and *Staphylococcus* biofilms in urinary catheters. The studies were extended to a *Pseudomonas* chronic pneumonia lung infection and *Staphylococcus* skin suture infection model in mice. The biofilm infections in mice, treated with shock waves became susceptible to antibiotics, unlike untreated biofilms. Mice exposed to shock waves responded to ciprofloxacin treatment, while ciprofloxacin alone was ineffective in treating the infection. These results demonstrate for the first time that, shock waves, combined with antibiotic treatment can be used to treat biofilm infection on medical devices as well as *in situ* infections.

Shock wave is a high energy wave travelling at supersonic speed, which is characterized by an abrupt change in pressure, temperature and density of the traversing medium. Unlike pressure wave, a shockwave is a single event of energy dissipation thus; no frequency is associated with it. They are generated in nature whenever the different elements in a fluid approach one another with a velocity higher than the local speed of sound at the present temperature. The unique character has widened the landscape of shock wave applications. Shock waves initially utilized in Aerospace research have found importance in bioengineering where extracorporeal shock waves are used as a non-invasive therapy in lithotripsy, wound healing and for treating avascular necrosis^1^,^2^,^3^. Previously we have used shock waves for vaccination, bacterial transformation and for drug delivery^4^,^5^,^6^,^7^. A few reports suggest that laser generated and extracorporeal shock waves can cause damage to the biofilm *in vitro*^8^,^9^,^10^,^11^,^12^, however the effects of shock waves on biofilms and the use of these shock waves as a therapy has not been reported.

It is now recognised that many infections are initiated from biofilms^13^,^14^. The US Centers for Disease Control and Prevention and National Institutes of Health have estimated that between 65–80% of infections are caused from biofilms^15^. A biofilm is an accumulation of microorganisms embedded in a Polymeric substance matrix that is adherent to a solid biologic or non-biological surface^13^,^14^. Bacteria contained within biofilms show resistance to antibiotics that is non-intrinsic. When the bacteria are released from the biofilm for planktonic growth, these same bacteria may be inherently sensitive to concentrations 1,000–10,000-fold less than those required to kill bacteria contained with the biofilm^16^.

Biofilms can be formed on most medical devices including urinary catheters, central venous catheters, peritoneal dialysis catheters, intrauterine devices, endotracheal tubes, prosthetic joints, voice prosthesis, mechanical heart valves and pacemakers^17^,^18^,^19^,^20^ by a wide variety of bacteria including *Escherichia coli*, *Pseudomonas aeruginosa and Staphylococcus aureus*. Contamination could be avoided in some of the devices by providing maximal sterile condition during insertion^21^.

Many pathogenic bacteria can form biofilms in or on tissues; these *in situ* biofilms cause inflammation and tissue damage^22^. These biofilms may form on surface of tonsils^23^ and on respiratory tract surfaces e.g. the paranasal sinuses^24^ etc. Planktonic bacteria released from these biofilms can intermittently or continually disperse to cause chronic infections, or infections at sites distal to the biofilm.

Here, we have successfully demonstrated the use of two different shock wave generators for the disruption of biofilms formed *in vitro* as well as *in vivo* conditions. The biofilms formed by *Salmonella*, *Pseudomonas* and *Staphylococcus* on urinary catheter surfaces have been exposed to shock waves produced by a hand-held device. A diaphragmless shock tube has been used for *in vivo* treatment of *Pseudomonas* lung infection and *Staphylococcus* skin suture infection in mice. This is first time that, an *in vivo* study of shock wave treatment of biofilms has been presented. The shock waves generated for the *in vivo* study are low amplitude and repeatable. The studies show that, in combination with antibiotic therapy, shock waves have the potential to treat lung and skin infections caused by bacteria.

## Results

### Biofilm formation

**Figure 1:**
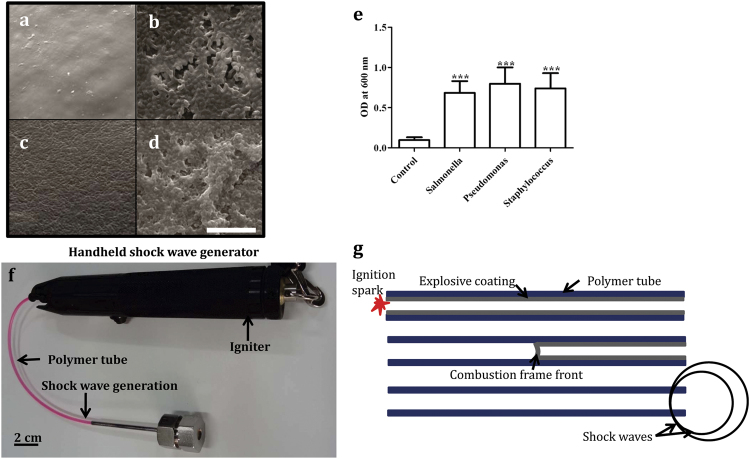
Biofilm formation on urinary catheters. (**a–d**) Scanning electron micrographs (SEM) of bacterial biofilms on sections of urinary catheter. The micrographs were taken at the same magnification and the scale bar in (**d**) is 10 μm. (**a**) Untreated catheter surface. (**b**) Catheter surface with *S.* Typhimurium biofilm. **(c)** *P. aeruginosa* biofilm. (**d**) *S. aureus* biofilm. (**e**) Crystal violet staining of biofilms caused by the different bacteria on equal amounts of catheter substrate. Statistical significance was calculated using One-way ANOVA. Asterisks indicate statistical significance as follows: (***p < 0.001). Error bar–mean ± SD. (**f**) Photograph of hand held shock wave generator. (**g**) Schematic of hand held shock wave generator. The igniter is used for the ignition of the polymer tube by a spark generated by electrodes, explosive coating undergoing combustion and the combustion flame front travelling at 2000 m/s, and shock waves emanating from the open end of the polymer tube.

Biofilms were grown on different substrates including plastic tubes and urinary catheters, using Gram positive and Gram negative bacteria including *Salmonella enteric sero*var Typhimurium (*S.* Typhimurium), *Pseudomonas aeruginosa* (*P. aeruginosa*) and *Staphylococcus aureus* (*S. aureus*). The extent of biofilm formation on urinary catheters was determined using scanning electron microscopy (SEM) and crystal violet staining (Fig. 1). All the tested bacteria formed biofilms under the conditions used. Biofilms were grown in the presence of urine on urinary catheters as determined by SEM (Fig. 1a–d) and the increase in biomass attached to the catheter surface was seen by crystal violet staining for biofilms^25^ (Fig. 1e).

### Generation of shock waves using hand-held device

For the *in vitro* studies, shock waves were generated using a hand-held shock wave generator (Fig. 1f)^4^,^5^,^6^. When the polymer tube is ignited at one end, a combustion front travels through the length of the tube and shock waves are generated at the other end (Fig. 1g). A typical shock wave pressure signal measured head on at a distance of 20 mm from the end of the tube is shown in (Fig. S1). The energy in the shock wave emanating from the open end of the polymer tube is estimated to be around 1.25 J (Table 1)^26^. The various parameters of the hand held shock wave generator are tabulated in table1. This mode of shock wave generation is ideal for *in vitro* study as it is a simple way to handle this portable device. The polymer tube was inserted to a 2 ml screw cap tube to generate shock waves and disrupt the biofilm.

### Impact of shock waves on biofilms on tubes and catheters

**Figure 2:**
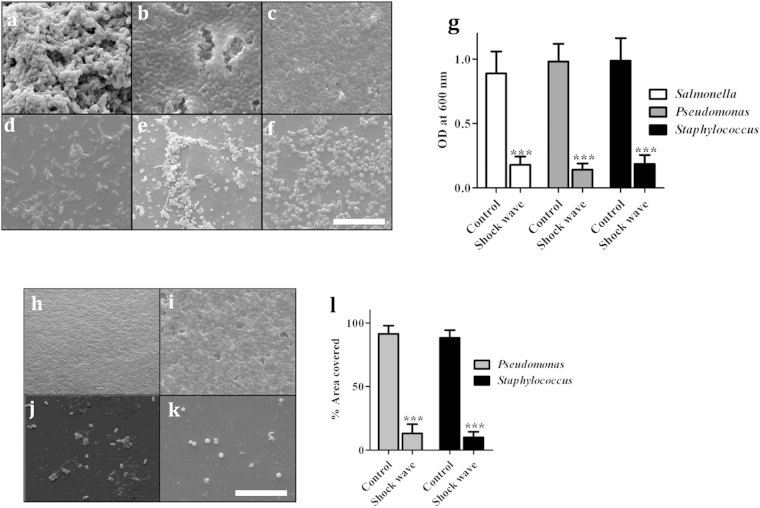
Effect of shock waves on biofilm formation. (**a–f**) SEM of bacterial biofilms grown on plastic microfuge tubes. (**a**) *S.* Typhimurium biofilm. (**b**) *P. aeruginosa* biofilm. (**c**) *S. aureus* biofilm. (**d**) *S.* Typhimurium biofilm after shock wave. (**e**) *P. aeruginosa* biofilm after shock wave. (**f**) *S. aureus* biofilm after shock wave. (**g**) Crystal violet staining of biofilms before and after shock wave treatment. (**h–k**) SEM of biofilms on urinary catheters before and after shock waves. (**h**) *P. aeruginosa* biofilm. (**i**) *S. aureus* biofilm. (**j**) *P. aeruginosa* biofilm after shock wave. (**k**) *S. aureus* biofilm after shock wave. (**l**) The area covered by the biofilm estimated from examining 50 SEM fields. Scale bar in 3f & 2k–10 μm. Statistical significance was calculated using One-way ANOVA. Asterisks indicate statistical significance as follows: (***p < 0.001). Error bar–mean ± SD.

The effect of a shock wave on biofilm maintenance was determined. The biofilms of *S.* Typhimurium (Fig. 2a), *P. aeruginosa* (Fig. 2b) and *S. aureus* (Fig. 2c) were initially grown in plastic screw cap tubes. SEM revealed that the biofilm grew efficiently on the plastic surface (Fig. 2a–c) but that examination of tubes with biofilms subjected to a shock wave revealed that the shock waves had greatly reduced the biofilm content, evidenced by SEM images (Fig. 2d–f) and in crystal violet assays (Fig. 2g). When *P. aeruginosa* (Fig. 2h) and *S. aureus* (Fig. 2i) biofilm were grown in urinary catheters similar reduction was observed upon exposure to shock waves (Fig. 2j,k). Manually calculating the areas in SEM images revealed that upon exposure to shock waves the biofilm was disrupted (Fig. 2l). While the shock wave reduced the biofilm, it did not reduce the viability of the bacteria (Fig. S2) and the bacteria were only released from the surface of the tube (Fig. S3). This data supports the observed reduction in the biofilm biomass by CV staining or SEM. Catheter biofilms grown in the presence of human or bovine urine showed equivalent levels of biofilm production, and reductions, through exposure to shock waves (Fig. S4).

### Increased sensitivity of biofilm bacteria treated with shock waves to antibiotics

**Figure 3:**
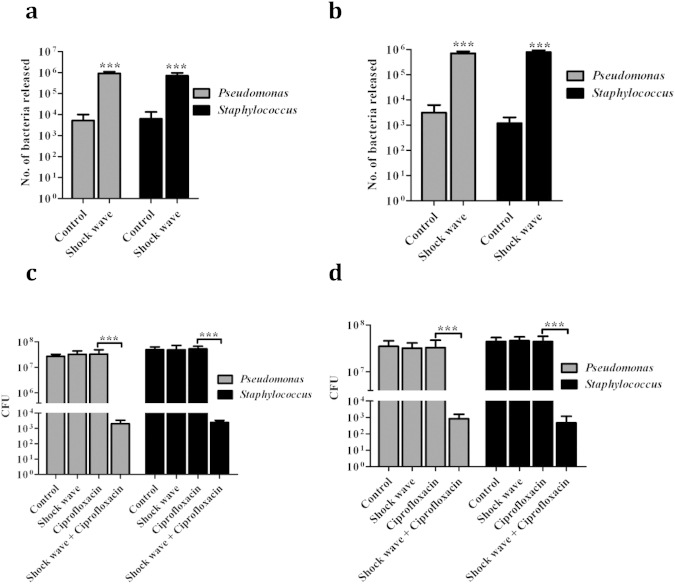
Antibiotic sensitivity of bacteria released by shock wave treatment. Catheter sections with biofilms of *P. aeruginosa* or *S. aureus* formed in bovine (**a**) or human (**b**) urine were washed and placed in PBS. After shock wave exposure, the PBS was plated to check the release of the bacteria from the biofilm. The control histograms were not treated with a shock wave. Catheter sections with biofilms formed in bovine (**c**) or human (**d**) urine were also treated with a shock wave, or left untreated, then incubated with 4 μg/ml ciprofloxacin. After 6 h, the catheter samples was washed, sonicated in a bath sonicator to release all bacteria, and plated in LA to estimate the number of viable bacteria. Control samples in c and d were exposed to neither shock waves nor ciprofloxacin. Statistical significance was calculated using One-way ANOVA. Asterisks indicate statistical significance as follows: (***p < 0.001). Error bar–mean ± SD.

Urinary catheters with biofilms were exposed to shock waves and the bacteria released from the catheters into bacterial media or urine were enumerated by viable colony count (Fig. 3). These results revealed that increased numbers of bacteria were released into the media by shock wave mediated biofilm dispersal (Fig. 3a,b), independent of the source of urine used to grow the biofilm. To determine whether this enhanced dispersal led to increased sensitivity of the biofilm to antibiotic treatment, biofilms in microfuge tubes and catheters were exposed to shock waves, incubated with 4 μg/ml ciprofloxacin for 6 h, the biofilm fully dispersed using a bath sonicator, and plated for viable count. The results showed that, while biofilms were structurally resistant to the antibiotic, the bacteria became susceptible to antibiotic after treatment with the shock waves (Fig. 3c,d). The use of the shock wave increased the biofilm community’s sensitivity to antibiotic by 100 to >1,000-fold. These data were replicated using biofilms formed in microfuge tubes (Fig. S5).

### Use of shock waves to reduce biofilm-mediated infection *in vivo*

#### *P. aeruginosa* pneumonia model

**Figure 4:**
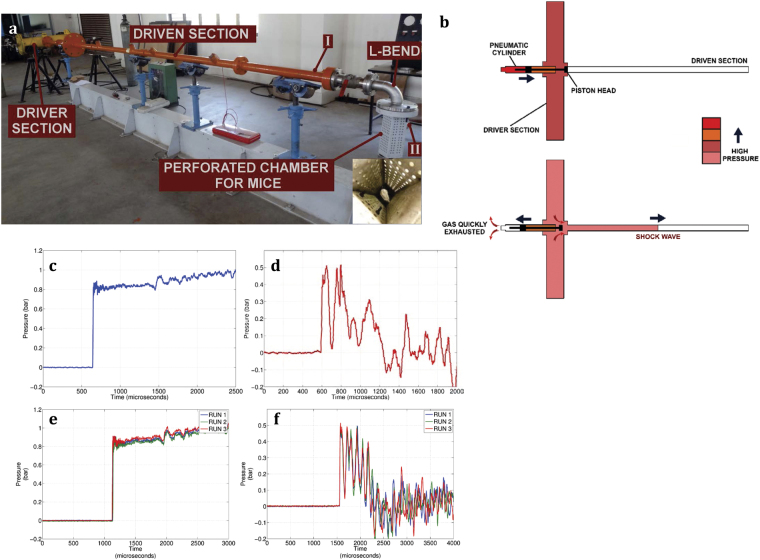
The diaphragmless shock wave generator and the pressure profile. (**a**) Photograph of diaphragmless shock wave generator/Diaphragmless shock tube (DST). The length of the driven section is 6.064 m. The L- bend which attenuated the shock wave by 30%. The mice were kept in the perforated chamber (insert) during shock wave treatment. (**b**) Schematic and working principle of DST. Pressure signal at the end of the shock tube (**c,e**) and inside the perforated chamber (**d,f**) was measured by mounting a pressure transducer at the respective places.

The extracorporeal shock waves generated using the hand-held shock wave generator are of high energy and are localized to a small area. Performing *in vivo* studies using the same device might cause tissue damage. This holds true even for other devices that are used to generate shock waves for biological applications, like lithotripters. Therefore, for the *in vivo* studies we have used a diaphragmless shock tube (DST) which generates low energy shock waves and the area of impact is larger so as to expose the entire body of the mouse to shock waves^27^. A conventional shock tube is a simple device having two sections, a driver section and driven section, separated by a metal diaphragm. In the case of DST, the metal diaphragm is replaced by a pneumatic cylinder (Fig. 4a,b).

The use of a pneumatic cylinder in place of metal diaphragms reduces the time between successive runs, as it does not require the replacement of a metal diaphragm for each run. Using the diaphragmless shock tube, shock waves of required strength (even as low as 0.8 bars over pressure which is required for our experiment) can be generated with high repeatability (Fig. 4c,e). Mice were housed in a perforated chamber that is mounted with a L-shaped bend at the end of the shock tube (Fig. 4a). The L-bend prevents the animals from entering into the shock tube. Also, the perforated chamber ensures that there are no reflections of the incident shock wave. The shock wave travelling through the L-shaped bend undergoes an attenuation of approximately 30% in strength^28^,^29^. The various parameters of this shock tube are shown in table 1.

For the biological experiments, the diaphragmless shock tube was operated with a driver section pressure (P4) of 5 bars and driven section (P1) kept at atmospheric pressure (1 bar). The pressure jump obtained behind the shock wave (P2) was ~ 0.8 bar at the end of the shock tube with high repeatability (Fig. 4c,e). This pressure is measured at the end of the shock tube before the L-shaped bend. The energy contained in the incident shock wave is calculated using the method suggested by Wang *et. al*.^30^. The energy in the incident shock wave is calculated to be 205 mJ/mm^2^. The mice in the perforated chamber experienced a peak overpressure of 0.48 bar (Fig. 4f). A specific impulse of 18.21 Pa.s/m^2^ was felt by the mice in the chamber, which is calculated by finding the area under the curve.

Mouse model of chronic pneumonia was used for *Pseudomonas* infection^31^. Mice (10 mice/group) were intranasally infected with 1 × 10^7^ CFU *P. aeruginosa* per mouse. The mice were killed 3 days after infection and the number of bacteria in the lungs was determined using viable count of homogenised lung tissue (Fig. S6a). The presence of bacteria and the formation of biofilm in lung were confirmed by SEM (Fig. S6b).

**Figure 5:**
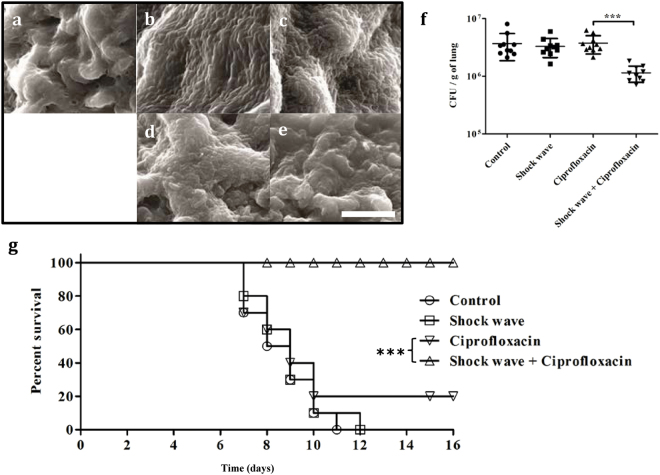
Use of shock wave adjunct therapy in the treatment of murine *P. aeruginosa* lung infection. BALB/c mice were infected with agarose beads coated with *P. aeruginosa* for 3 days before treatment with a daily shock wave, with and without daily ciprofloxacin therapy for a further 3 days. (**a–e**) The mice were killed and the lungs examined by SEM. (**a**) Uninfected mice. (**b**) Mice infected with *P. aeruginosa*. (**c**) Mice infected with *P. aeruginosa* and treated with ciprofloxacin (2.5 mg/kg, via intravenous delivery). (**d**) Mice infected with *P. aeruginosa* and treated with shock wave therapy alone. (**e**) Mice infected with *P. aeruginosa* and treated with a shock waves and ciprofloxacin. Scale bar in 4E–10 μm. (**f**) The number of bacteria in the lungs were determined by plating the homogenized lung tissue after 3 days of treatment. The Statistical significance was calculated using Mann-Whitney test. Asterisks indicate statistical significance as follows: (*p < 0.01), (***p < 0.001), ns–not significant. Error bar–mean ± SD. (**g**) The survival of mice infected with *P. aeruginosa* for 3 days, then treated with ciprofloxacin with and without shock waves for 5 days (day 0 in g). The animals in g were examined twice daily. The Statistical significance was calculated using Log-rank test. Asterisks indicate statistical significance as follows: (***p < 0.001)

Mice that were infected intranasally with *P. aeruginosa* were treated with ciprofloxacin alone, with shock waves alone, or with a combination of shock wave and ciprofloxacin for 3 days. The mice were killed after 3 days of treatment and the effect of treatment on lung tissue was examined by SEM. No bacteria were found in the uninfected mice, a clear biofilm formation was observed in the infected mice and reduced number of bacteria were found in the treatment group with shock wave and antibiotic (Fig. 5a,e). The numbers of bacteria in lung homogenates were determined by viable count (Fig. 5f). Though the number of bacteria observed in SEM of tissues from mice treated with shock waves alone (Fig. 5d) was greatly reduced compared with control mice (Fig. 5b), there was no reduction in the number of bacteria found in tissue homogenates by viable count (Fig. 5f). The viable count results showed that *P. aeruginosa* infection in mice, initiated by intranasally delivered agarose beads, was not sensitive to daily treatment with ciprofloxacin. In contrast, the combination of shock wave and ciprofloxacin treatments significantly reduced the number of bacteria in the lung, determined by viable count (Fig. 5f).

The impact of shock wave therapy on the survival of mice with *P. aeruginosa* pneumonia was also examined. At the end of the therapy (Day 0 in Fig. 5g), groups of 8 mice were examined twice daily to determine when they became moribund. Infected mice given shock wave therapy alone or left untreated died within 10 days whereas only 2 mice treated with ciprofloxacin alone survived. All mice provided ciprofloxacin therapy and shock wave treatment survived. From these data it is evident that shock waves generated from the diaphragmless shock tube can be used to treat lung biofilm infection without any damage to the lung tissue. Further detailed studies are required to confirm the safety of using shock waves *in vivo* for other organs.

#### *S. aureus* infected suture model

**Figure 6:**
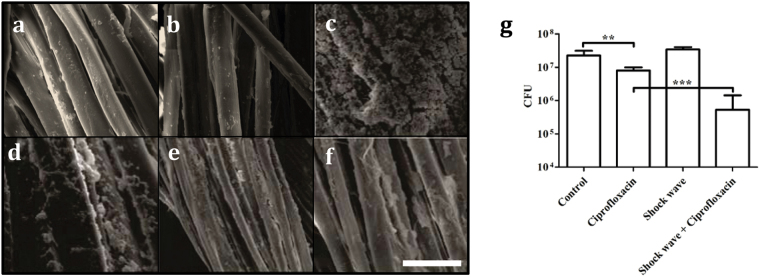
Use of shock wave adjunct therapy in the treatment of murine suture associated *S. aureus* infections. BALB/c mice were provided abdominal sutures after 3 days the sutures were removed. SEM of untreated sutures revealed that sterile sutures did not develop obvious biofilms (**a**). (**b**) SEM of Sterile suture treated with shock wave. Mice were also given sutures where the surgical wire was pre-incubated with *S. aureus* (**c-f**). After 3 days the mice were killed and the sutures examined by SEM. The excised *S. aureus* biofilm-coated sutures were left untreated **(c)**, treated *ex vivo* with ciprofloxacin alone (4 μg/ml for 6 h) (**d**), shock wave therapy alone **(e)** or a combination of ciprofloxacin and shock wave (**f**). Scale bar in e–10 μm. **(g**) The bacteria were enumerated in the suture with or without any treatment by homogenizing by ultrasonication and plating. Statistical significance was calculated using One-way ANOVA. Asterisks indicate statistical significance as follows: (**p < 0.005), ( p < 0.001). Error bar–mean ± SD.

Anaesthetized mice (5 mice/group) were shaven of abdominal fur and provided surgical wire sutures. Sterile sutures removed after 3 days and examined by SEM did not carry obvious biofilms (Fig. 6a). Sterile suture exposed to shock waves did not show any defects or damage (Fig. 6b). Mice were also administered sutures that were pre-incubated in TSB broth containing *S. aureus* (Fig. 6c,f). After 3 days, the sutures were removed and the formation of biofilms on the sutures was observed by SEM. The excised *S. aureus* contaminated suture wires were treated *ex vivo* with ciprofloxacin (4 μg/ml for 6h) alone (Fig. 6d), shock waves alone (Fig. 6e), or a combination of shock wave treatment ciprofloxacin (4 ug/ml for 6h). The remaining sutures not used in SEM were homogenized and the number of bacteria determined by viable count (Fig. 6g). The results show that there is a significant reduction in the suture wires treated with ciprofloxacin compared with control infected sutures by SEM (Fig. 6d) and viable count (Fig. 6g). The biofilm associated with sutures was reduced further when the excised suture wire was treated with shock waves and ciprofloxacin (Fig. 6f,g). Shock wave therapy alone reduced the presence of bacteria as observed in SEM (Fig. 6e), but not by viable count (Fig. 6g).

**Figure 7:**
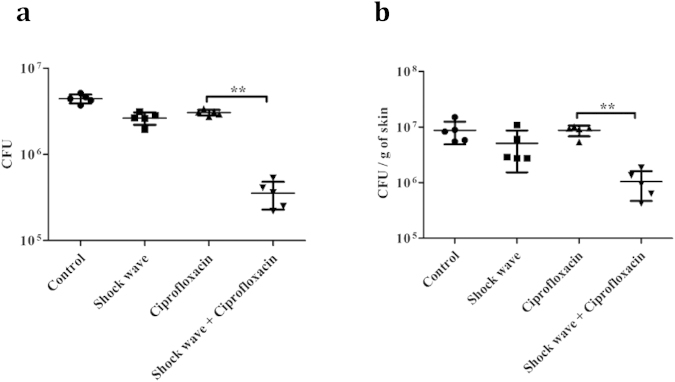
Use of shock wave adjunct therapy in the treatment of murine *s*uture-associated *S. aureus* infections. BALB/c mice were provided abdominal sutures and the sutures were subsequently infected with *S. aureus.* The mice were left untreated (control), treated with shock wave therapy alone, ciprofloxacin alone (i.v. 2.5 mg/kg daily) or a combination of ciprofloxacin and shock wave. After 3 days of treatment, the sutures (**a**) and surrounding skin (**b**) were removed independently and homogenised and the bacteria were enumerated by viable count. The Statistical significance was calculated using Mann-Whitney test. Asterisks indicate statistical significance as follows: (**p < 0.005). Error bar–mean ± SD.

To determine whether this combination of shock wave therapy and antibiotic treatment could reduce the biofilms associated with infected sutures *in vivo*, an abdominal incision was made and the incision closed with suture wire. The suture site was then infected with *S. aureus* and the mice were treated with ciprofloxacin intravenously with or without shock wave exposure using diaphragmless shock tube. After treatment for 3 days, the number of bacteria on the surgical wire and surrounded skin tissue were analysed by excising either the sutures or the adjacent skin, homogenising the sample, and plating the samples for viable count of the bacteria (Fig. 7). Control animals received no sutures and *S. aureus* infection without further treatment. The bacterial numbers associated with sutures (Fig. 7a) or surrounding skin (Fig. 7b) were equivalent in all mice, except those that received combination shock wave and antibiotic therapy.

## Discussion

In 1982, Chaussey developed extracorporeal shock wave lithotripsy (ESWL) by creating high pressure at a focal point by shock focusing^32^. To disintegrate the kidney stones, the shock wave should generate at least 80bar since the yielding stress of kidney stones is 80bar at maximum^33^.

Though shock waves have been used clinically for many years, the high pressure profile and cavitation effects of shock waves can lead to the damage of the tissues^34^. The extracorporeal shock waves are generated by focussing at a particular site and hence the energy is very high at the localized area and hence the tissue damage is possible. Tissue damage will depend on the shock wave energy density, number of shock waves and shock wave coupling. Hence the threshold limit should be used to avoid any tissue damage or bleeding^35^. Shock wave lithotripsy carried out in rabbits is found to cause haemorrhage, edema, congestion, inflammation, loss of normal structure and epithelial desquamation in all areas of the pleural tissue. The reason for the lung injury is attributed to the tissue-air interphase as lungs consist of alveolar sacs^36^. In 1987, lung hemorrhage was observed in dogs when they were exposed to shock waves with 100 bar overpressure but not with 20 bar^37^. The severity of the damage depends on several factors, such as shock wave energy density, type of tissue, number of shock waves, etc. In our experiments, the shock wave energy is distributed to the whole body, since, we have used unfocussed, low energy shock waves to disrupt the biofilm and we did not observe lung tissue damage caused using this method. Further detailed studies are necessary to understand the other deleterious effects of shock waves on other organs.

Biofilm formation occurs through different phases such as surface conditioning, attachment and colonization. Surface conditioning involves the adsorption of organic and inorganic nutrients, which facilitate bacterial attachment. Initial attachment of the bacteria to the surface may be reversible involving Brownian movement and weak forces between the surfaces or may involve ligands (e.g. bacterial fimbriae) binding to cognate receptors^13^,^14^. Regardless, the early interactions can soon become irreversible due to the production of extracellular polymeric substances (EPS)^38^. As the biofilm-associated bacteria grow and divide, the colony may form simple or complex tertiary structures^39^, which demonstrate increased resistance to antibiotics. There are multiple hypotheses to explain the phenotypic resistance observed including slow or incomplete penetration of the antibiotic into the biofilm, altered microenvironment through the depletion of nutrients or accumulation of waste products within the biofilm, and/or the formation of resistant phenotypes a result of an altered growth rate of the bacteria^38^.

Much of the recent research aimed at preventing biofilm formation is focused on using novel materials or catheters coated with antibiotics. Antimicrobial peptides coated implants have been developed to control the biofilm formation in these implants. While these approaches offer some promise, the architecture of biofilms where there is clear separation between the growing edge of the biofilm and the surface, may mitigate against success of this approach. Ultrasound has also been used to kill the biofilm mode of infection along with antibiotics^10^. Apart from this, photon-initiated photoacoustic streaming has been used to remove dental biofilm^11^.

We have used shock waves to disrupt biofilms in urinary catheters at physiological conditions, where the biofilms were grown in bovine or human urine. When the catheter-associated biofilms were treated with ciprofloxacin there was no reduction in the number of bacteria, whereas ciprofloxacin treatment together with shock wave therapy significantly reduced the number of bacteria that could be observed or detected by viable count. The shock waves were not antibacterial. It is hypothesized that the very brief exposure to the wave ruptured the polysaccharide matrix surrounding the biofilm, liberating bacteria and possibly increasing access to the antibiotic. Further experiments were carried out to determine the effect of shock waves on *in vivo* biofilms.

Mice intranasally administered *P. aeruginosa* developed biofilms in the lung after 3 days as seen in scanning electron microscopy (SEM) images. When these mice were treated with ciprofloxacin, there was no decrease in the bacterial counts in homogenised lung samples, but this bacterial burden was significantly decreased if the animals received antibiotic treatment combined with shock wave therapy. The shock wave therapy neither causes pathology to the lungs, as determined histologically (Fig. S6c–h) nor any weight loss (Fig. S6i). Very few mice survived without antibiotic treatment and they showed 10 to 20% weight loss, whereas, shock wave along with antibiotic treatment group mice recovered from initial 3 days of weight loss (Fig. S6i). Mice that received both antibiotic and shock wave therapy after challenge with agarose beads coated with *P. aeruginosa* survived, whereas animals that received antibiotics alone usually succumbed to infection.

Wound infections driven by biofilms associated with sutures and other closure devices are a major cause of nosocomial infection. It is estimated that 1–8% of the people who have undergone cardiac surgery develop wound site infections^40^. We have simulated this condition in mouse skin, where an incision was made and closed with surgical suture wire. The wire was either infected with *S. aureus* before insertion, or subsequently. SEM analysis that was a near complete disruption of biofilms formed on suture wires *in vitro* if the wires were excised and treated with shock waves *ex vivo*. If the animals carrying the sutures were subject to both shock wave and antibiotic therapy, and the degree of *S. aureus* infection of the suture wires, and the surrounding tissues, was reduced significantly. Consistent with observations that shock waves alone are not antibacterial, therapy using shock waves in the absence of antibiotics, like antibiotic therapy alone, was not effective in reducing the bacterial burden of suture sites contaminated with *S. aureus*. Here we have shown for the first time that a single shock wave pulse of 205 mJ/mm^2^ can be used to disrupt biofilm *in vivo*. Though, the energy in the shock wave is much higher than that of the shock wave generated by the hand-held device, the area over which the shock wave is spread is larger. Therefore, the energy per unit cross-section area is lower than that of the hand-held device. Also, the peak over-pressure of the shock wave is lower. The steady time duration of the shock wave is much longer in the case of the diaphragmless shock wave generator. All these parameters help in conducting the *in vivo* studies without injuring the different organs of the mice.

These results clearly indicate that shock waves can be used as an adjunct therapy to treat bacterial biofilm infection in medical devices and foreign body-associated infections. The data demonstrates, for the first time, that shock wave therapy can be used to treat biological biofilm infections in combination with antibiotic treatments. The combined use of shock wave and ciproflaxin, while yielding significant reduction in viable bacteria in *in vivo* tests, resulted in approximately 1 log in CFU reduction, leaving substantial viable bacteria, which might include resistant individuals. The effect of the shock wave on the generation of resistance is not known and requires further study.

The shock waves generated in the present study are completely different from the extracorporeal shock waves, which are routinely used for kidney stone disintegration in terms of the impulse and peak pressure amplitude. Since ESWL is being routinely practiced for treatment of unrelated medical conditions, shock wave assisted biofilm disruption could be used to treat especially recalcitrant biofilm infections in hospital settings. Since this methodology of shock wave generation and exposure to treat biofilm infections can easily be scaled up for human trials, the finding of this interdisciplinary study will have profound impact in therapeutic uses in the near future.

## Methods

### Bacterial strains and growth conditions

*Salmonella enteric* serovar Typhimurium (ATCC 14028s), *Pseudomonas aeruginosa* (PA01) and *Staphylococcus aureus* (ATCC 25923) were grown in Luria broth at 37 °C in a shaker at 180 rpm. The bacteria were maintained on Luria agar.

### Biofilm formation

#### Microfuge tubes

For *S.* Typhimurium biofilm formation, 1 × 10^4^ stationary phase cells were inoculated into 2 ml of Luria broth without NaCl and kept at 25 °C in static condition in 2 ml screw cap tubes, with or without cholesterol coating (1 mg). 100 μl of cholesterol dissolved in anhydrous ether (10 mg/ml) was taken in the microfuge tubes and the ether was allowed to evaporate to coat 1 mg of cholesterol in the microfuge tubes. Cholesterol coating was used to mimic the physiological gall bladder stone condition^41^. For *P. aeruginosa* and *S. aureus* biofilm formation, 1 × 10^4^ stationary phase cells were inoculated in 2 ml of tryptic soy broth and kept at 25 °C in static condition for better biofilm formation in 2 ml screw cap tubes.

#### Urinary catheters

Sterile cover slip or catheter (Foley catheters, Silicone coated latex, 8F–10F) (1 cm segment) was placed in a 24-well plate with either 1 ml of tryptic soy broth (TSB), bovine urine (filter sterilized) or human urine (filter sterilized). 1 × 10^4^ stationary phase cells of *S.* Typhimurium, *P. aeruginosa* and *S. aureus* were inoculated in the 24-well plate and kept at 25 °C in static condition for 72 h. The respective media was changed every 24 h.

#### Surgical sutures

Overnight grown *S. aureus* culture in tryptic soy broth (TSB) were inoculated (1 × 10^7^ bacteria) into 1 ml of fresh TSB in a 24 well plate. 5 mm sections of sterile surgical sutures (Mersilk^TM^, NW5079–non-absorbable and made of fibroin protein, suture size: 5-0) were placed in the wells. The plates were incubated at 25 °C in static condition with regular change of fresh TSB at 24 h intervals.

### Shock wave generation using hand held device

Shock waves were generated using high melt explosive [octahydro-1,3,5,7-tetranitro-1,3,5,7-tetrazocine] and traces of aluminium coated polymer tube^4^,^5^,^6^. Briefly, when the explosive coated polymer tube was ignited from one end of the tube, shock waves are produced at the other end of the tube (Fig. 1f,g). These shock waves are ideal for *in vitro* experiments but not for *in vivo* experiments as the dimensions of the specimen are bigger and the entire body of the mouse needs to be exposed to the shock wave (Table 1). Impulse per unit area (I) was calculated by using the formula,


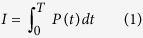


Where, ‘T’ represents the steady time of the shock wave, ‘P (t)’ is the pressure experienced by the sample as a function of time.

### Crystal violet (cv) staining of biofilms

Microfuge tubes containing biofilm on the inner walls of the tubes and biofilm on the surface of the catheters were confirmed by crystal violet staining^25^. After 72h of incubation at static condition, the tubes/catheters were washed with phosphate buffered saline (PBS) three times to remove the planktonic cells. The tubes/catheters were dried and stained with 1% (w/v) crystal violet for 15 minutes at room temperature. After three PBS washes, the bound crystal violet was solubilized with 200 μl of absolute ethanol. The optical density was determined at 600 nm (SpectraMax 340PC, Molecular Devices).

### Shock wave assisted disruption of biofilms

After 72 h of incubation under static conditions, the tubes, catheters or sutures were washed three times with PBS to remove the planktonic cells. The tubes were filled with 1.5 ml of PBS and exposed to shock waves. In case of catheters and sutures, the samples were placed in 2 ml screw cap tube containing 1.5 ml PBS. 2 mm hole was made in the 2 ml screw cap in a sterile condition and the polymer tube was inserted without touching the liquid or sample and triggered for shock wave generation (Supplementary video 1). The generation of blast sound upon shock wave exposure assures that the materials were exposed to shock waves. The tubes, catheters and surgical suture wires were further analyzed by CV staining, scanning electron microscopy (SEM), CFU measurement and antibiotic sensitivity assay to validate the disruption caused by shock waves.

### Scanning electron microscopy (SEM)

Catheters, surgical sutures and cover slips with biofilm were fixed with 2.5% (v/v) glutaraldehyde and the samples were dehydrated with increasing concentrations of ethanol for 2 min each. The samples were stored in vacuum until use. Prior to analysis by Field emission SEM (FEI-SIRION, Eindhoven, Netherlands), the samples were subjected to gold sputtering (JEOL JFC 1100E Ion sputtering device).

### Antibiotic sensitivity assay

After biofilm shock wave treatment (as described earlier), the samples were exposed to 4 μg/ml of ciprofloxacin under shaking conditions at 180 rpm, 37 °C for 6 h, washed with PBS and homogenized by sonication for 10 minutes in a bath sonicator and plated on LA^42^. The untreated biofilm samples were used as controls.

### Release of bacteria

After shock wave exposure to biofilm in tubes and in catheters, the media (PBS) containing the sample was transferred to a new microfuge tube, homogenized by sonication for 5 minutes in a bath sonicator and plated on LA to determine the number of bacteria released from the biofilm. The media (PBS) from the untreated biofilm samples were also processed similarly and used as controls.

### *P. aeruginosa* lung infection mouse model

BALB/c mice bred and housed at the Central Animal Facility of Indian Institute of Science (IISc) were used for all the *in vivo* experiments. The mice used for the experiments were 6–8 weeks old. All procedures with animals were carried out in accordance with the Institutional rules for animal experimentation. The Institutional Animal Ethics Committee (Registration No: 48/1999/CPCSEA) approved all animal experimental protocols and the National Animal Care Guidelines were strictly followed. Chronic pulmonary infection in mice was induced by agarose bead infection method as described elsewhere^31^. Briefly, adult BALB/c mice were infected by intranasal injection of bacterial beads that were produced by mixing 1 ml of overnight culture of *P. aeruginosa* with 9 ml of PBS and 25 ml of 2% (w/v) agarose. The mixture was stirred continuously after adding 500 ml of mineral oil at 50 °C until the temperature reach RT. The agarose beads were washed once with 0.5% deoxycholic acid, sodium salt (SDS) in PBS, once with 0.25% SDS, and four times with PBS. The number of viable bacteria was enumerated and the beads were stored for not more than 48 h at 4 °C.

BALB/c mice (10 mice/group) were intranasally infected with 50 μl of 1:10 dilution bead slurry. On day 3 post infection, different cohorts of mice were treated with ciprofloxacin alone (2.5 mg/kg–intravenous injection–once per day), shock wave treatment alone (once per day), or shock wave (once per day) and ciprofloxacin treatment (2.5 mg/kg–intravenous injection–once per day) for 3 days. The mice were killed after 3 days, and the lungs was aseptically removed, weighed and homogenized in sterile PBS. The homogenate was plated using serial dilutions onto LB agar to determine the bacterial load. A small part of the lung was fixed with 2.5% (v/v) glutaraldehyde and processed with increasing concentrations of alcohol (10, 20 30, 50, 70, 80, 90 and 100%) and analysed with SEM as described. For survival assay, same treatment was given (n = 8 mice/group) for 5 days and the mice were monitored twice daily for 20 days for morbidity and mortality.

### *S. aureus* skin suture infection mouse model

The abdominal fur of BALB/c mice (5 mice per group) was removed with a razor and the mice were anesthetized by intraperitoneal injection of a mixture of ketamine (100 mg/kg) and xylazine (10 mg/kg). A small incision was made in the abdomen, which was subsequently closed using surgical suture (Mersilk^TM^, NW5079). 10 μl of overnight grown *S. aureus* culture (1 × 10^7^) was applied to the site and allowed to dry. Three days after infection, different groups of mice were treated with ciprofloxacin alone (2.5 mg/kg–intravenous injection–once a day), shock wave treatment alone (once per day), or shock wave (once per day) ciprofloxacin treatment (2.5 mg/kg – intravenous injection–once a day), for 3 days. The mice were killed after 3 days, the suture wire and the surrounding skin was dissected and homogenized in sterile PBS. The homogenate was plated in serial dilutions on Vogel-Johnson agar to determine the bacterial load in the sample.

### Diaphragmless shock tube for *in vivo* experiments

Experiments were carried out using a diaphragmless shock wave generator^27^. The diaphragmless shock tube has internal diameter of 50 mm and employs a fast acting pneumatic valve instead of aluminium diaphragms. The driver section volume is 0.0454 m^3^ and the driven section length is 6.064 m. Initially, the pneumatic cylinder is pressurized to facilitate the forward motion of the piston head. The piston head seals the junction between the driver and the driven section. The driver section is filled with a high pressure (P4) while the driven section is maintained at a low pressure (P1). When the gas at the rear end of the pneumatic cylinder is suddenly exhausted, the piston head moves back at a high speed (~5 ms) allowing the high pressure driver gas to enter the driven section. This generates a shock wave in the driven section with an overpressure P2. The amplitude of pressure P2 can be controlled by the initial pressure conditions in the driver and driven section i.e. P4 and P1.

When used with mice, the animals are housed in a perforated chamber that is mounted with a L-shaped bend at the end of the shock tube (Fig. S4a). Impulse per unit area (I) can be calculated by using the equation (1).

### Statistical analysis

All the *in vitro* and *in vivo* experiments were performed in triplicates. One way ANOVA, Mann-Whitney tests and Log-rank test were performed using GraphPad Prism 5 software.

## Additional Information

**How to cite this article**: Gnanadhas, D. P. *et al.* Successful treatment of biofilm infections using shock waves combined with antibiotic therapy. *Sci. Rep.* **5**, 17440; doi: 10.1038/srep17440 (2015).

## Supplementary Material

Supplementary Information

Supplementary Datasets

Supplementary Video 1

## Figures and Tables

**Table 1:** Comparison of various parameters of hand held shock wave generator and diaphragmless shock tube.

Device	Hand held shockwave generator tube	Diaphragmlessshock tube
Type of experiment	*In vitro*	*In vivo*
Diameter of the tube	2 mm	50 mm
Cross sectional area	3 mm^2^	1963 mm^2^
Energy of the shock wave	1.25 J	403 J
Energy per unit area at the exit	416 mJ/mm^2^	205 mJ/mm^2^
Peak over pressure (Standoff distance)	3.24 bar (20 mm)	0.48 bar (290 mm)
Duration of peak pressure	50 μs	600 μs
Impulse per unit area	0.9545 PaS	18.21 PaS
