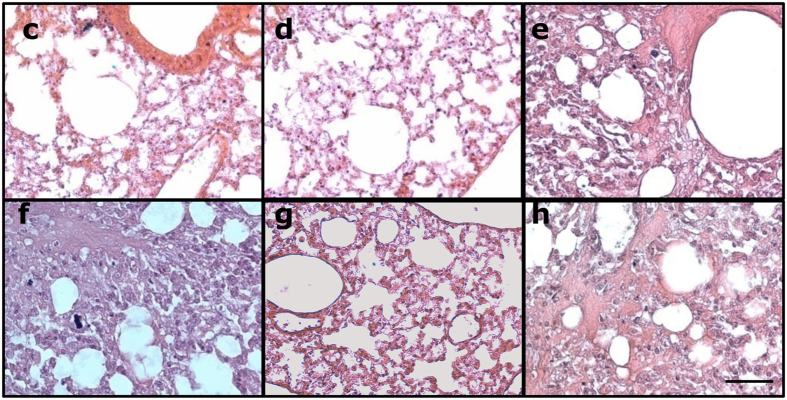# Corrigendum: Successful treatment of biofilm infections using shock waves combined with antibiotic therapy

**DOI:** 10.1038/srep46929

**Published:** 2018-01-08

**Authors:** Divya Prakash Gnanadhas, Monalisha Elango, S. Janardhanraj, C. S. Srinandan, Akshay Datey, Richard A. Strugnell, Jagadeesh Gopalan, Dipshikha Chakravortty

Scientific Reports 5: Article number: 1744010.1038/srep17440; published online: 12 10 2015; updated: 01 08 2018

Figure S6 in the Supplementary Information contains errors. Due to misfiling of data the figure was prepared incorrectly. The image in figure S6g corresponds to the incorrect experimental group: ‘Mice infected with *P. aeruginosa* and treated with ciprofloxacin.’ rather than ‘Mice infected with *P.aeruginosa* and treated with shock wave therapy alone.’ The correct figure 6Sc-h appears below as Figure 1.

## Figures and Tables

**Figure 1:**